# Prenatal Exposure to Polychlorinated Biphenyls and Postnatal Growth: A Structural Analysis

**DOI:** 10.1289/ehp.8488

**Published:** 2005-12-29

**Authors:** Matthew R. Lamb, Sylvia Taylor, Xinhua Liu, Mary S. Wolff, Luisa Borrell, Thomas D. Matte, Ezra S. Susser, Pam Factor-Litvak

**Affiliations:** 1 Department of Epidemiology and; 2 Department of Biostatistics, Mailman School of Public Health, Columbia University, New York, New York, USA; 3 Department of Community and Preventive Medicine, Mount Sinai School of Medicine, New York, New York, USA; 4 New York Academy of Medicine/Center for Urban Epidemiologic Studies, New York, New York, USA

**Keywords:** adverse effects, cohort studies, environmental exposure, growth, height, human, polychlorinated biphenyls, prenatal exposure, weight

## Abstract

Normal endocrine function *in utero* and early in childhood influences later height and weight attainment. Polychlorinated biphenyls (PCBs) are persistent environmental contaminants with suspected endocrine-disrupting properties. PCBs may mimic or inhibit hormone and endocrine processes based in part on their structural configuration, with non-*ortho*-substituted PCBs having a coplanar orientation and *ortho*-substituted PCBs becoming increasingly noncoplanar. Coplanar and noncoplanar PCBs have known differences in biologic effect. Animal studies link prenatal PCB exposure to adverse birth and early-life growth outcomes, but epidemiologic studies are conflicting. We examined whether prenatal exposure to PCBs, categorized by their degree of *ortho*-substitution, affected childhood height and weight attainment in 150 children (109 boys and 41 girls) with African-American mothers born at the Columbia-Presbyterian Hospital from 1959 through 1962. Stratifying by sex, we used regression models for repeated measures to investigate associations between maternal levels of PCBs and height and weight through 17 years of age. Maternal levels of *ortho*-substituted PCBs were associated with reduced weight through 17 years of age among girls but not among boys. Tri-*ortho*-substituted PCBs were marginally associated with increased height in boys. Although limited by sample size, our results suggest that prenatal exposure to PCBs may affect growth, especially in girls, and that *ortho*-substitution is an important determinant of its effect on growth.

Proper functioning of maternal and fetal hormone and endocrine systems is essential to ensure a properly developing fetus. Suboptimal endocrine functioning *in utero* can have lifelong effects on growth and development (Fowden and Forhead 2004). Sex hormones, such as testosterone and estrogen, stimulate growth in children (Jacklin et al. 1994). Estrogens are responsible for the female pubertal growth spurt and the development of secondary sex characteristics and have a primary role in the regulation of bone density, whereas both estrogens and androgens are important for growth in boys (Bilezikian 2001; Cutler 1997; Frank 2003). Further, the levels of circulating hormones differ between boys and girls in the first 6 months of life, suggesting their early importance in setting up differential development by sex (Jacklin et al. 1994).

Endocrine function may be altered by exposure to specific environmental chemicals, such as polychlorinated biphenyls (PCBs). As a class consisting of 209 congeners with various numbers of chlorine substitutions, PCBs are persistent environmental pollutants detectable in nearly all human populations. The degree of substitution of hydrogen atoms on the biphenyl ring by larger and negatively charged chlorine ions creates structurally distinct compounds. PCBs with no substitutions at the *ortho*-position (closest to the bond connecting the two phenyl rings) are coplanar, whereas those with substitutions become increasingly noncoplanar. Several reviews suggest that the planarity in part determines their biologic effect (Fischer et al. 1998; McKinney and Waller 1994; Safe 1993).

Evidence suggests that prenatal exposure to PCBs affects *in utero* and postnatal growth. Studies in rhesus monkeys (Allen et al. 1980; Barsotti and Van Miller 1984) and rats (Brezner et al. 1984; Overmann et al. 1987) link prenatal exposure to PCBs with reduced birth weight. Other animal studies provide evidence of reduced postnatal weight (Morse et al. 1996) or decreased postnatal weight gain among offspring of mothers exposed to PCBs (Bushnell et al. 2002; Faqi et al. 1998; Hany et al. 1999; Mccoy et al. 1995). Some but not all epidemiologic research has shown associations between prenatal PCB exposure and reduced growth outcomes. Investigations in Japan and Taiwan into children of mothers heavily exposed to PCBs and polychlorinated dibenzofurans found evidence of intrauterine growth retardation and reduced growth until school age (Guo et al. 1994; Rogan et al. 1988), with a stronger association among girls (Guo et al. 1995; Yamashita and Hayashi 1985). Investigations looking at chronic, lower-dose exposures in Michigan (USA) (Blanck et al. 2002; Jacobson et al. 1990), the Netherlands (Patandin et al. 1998), and Sweden (Mazhitova et al. 1998) found associations between prenatal or perinatal exposure to PCBs and reduced growth outcomes at birth and in early life. However, a prospective investigation in North Carolina (USA) found no evidence of an association between PCB exposure and reduced growth through 14 years (Gladen et al. 2000; Rogan et al. 1986a, 1986b, 1987), and an investigation into prenatal exposure to PCBs and other organochlorines in the Ukraine found no effect of exposure on birth weight (Gladen et al. 2003). Another study measuring blood organochlorine concentrations in children at 8 years of age found no association between PCB levels and height, although it did find a dose–response relationship between exposure to dichlorodiphenyldichloroethylene (DDE) and reduced girls’ height (Karmaus et al. 2002).

In this pilot study we extend the previous investigations by classifying prenatal exposure to PCBs by their degree of *ortho*-substitution and explore the associations between these categories and childhood growth. Previous studies either used estimates of contaminated food consumption as a proxy measure for PCB exposure (Guo et al. 1994, 1995; Jacobson et al. 1990; Yoshimura 2003), measured only a few PCB congeners (Grandjean et al. 1997; Winneke et al. 1998), or treated PCBs as a single exposure (Jacobson et al. 1990; Rogan et al. 1986b). To our knowledge, only the Dutch cohort looked at both congener-specific exposure to PCBs and a grouped measure of exposure (based on toxic equivalencies to dioxin) (Patandin et al. 1998; Schantz et al. 2003). Other studies using congener-specific analysis are ongoing (Schantz et al. 2003), although to our knowledge none intends to classify exposure by degree of *ortho*-substitution. Here, we investigate whether prenatal exposure to PCBs is associated with reduced height and weight through 17 years of age. Specifically, we hypothesize that structural classes of PCBs, based on their degree of *ortho*-substitution, will have different effects on height and weight attainment, and that these effects will differ by sex.

## Materials and Methods

### Study design.

The mother–child pairs in this study represent a subset of the Columbia-Presbyterian cohort of the National Collaborative Perinatal Project (NCPP). The NCPP was initiated > 40 years ago to prospectively investigate the prenatal and familial antecedents of childhood disorders (Hardy 2003). Detailed information on the sampling strategy for this subset is provided elsewhere (Shaffer et al. 1985). Briefly, our subset consists of African-American children born between 1959 and 1962 whose mothers supplied written informed consent to participate in a study investigating the association between the presence of neurologic soft signs (abnormal motor or sensory function without evidence of a localized lesion) at 7 years of age and intelligence and psychiatric disorders in late adolescence (Pine et al. 1997; Shaffer et al. 1985). Sixty-three male and 27 female children with neurologic soft signs at 7 years of age were matched to children from the same cohort but without evidence of soft signs; 162 agreed to undergo psychiatric assessments at 17 years of age (Shaffer et al. 1985). Height and weight measures were taken at birth and 1, 4, 7, and 17 years of age, although the measures at 1 year of age were considered unreliable because of measurement error and were excluded from the analysis.

### Laboratory analyses.

Maternal blood sera obtained during the third trimester of pregnancy and stored at –20°C since collection (1959–1962) were analyzed for 24 PCB congeners, DDE, dichlorodiphenyltrichloroethane (DDT), and levels of serum cholesterol and triglycerides. There have been no reported thaws of the banked sera (Klebanoff M, personal communication), and organochlorine degradation under these conditions should be minimal (Trapp et al. 1984). Because PCBs have a long half-life in adipose tissue and because we did not expect levels to vary during pregnancy (Longnecker et al. 1999), the third-trimester concentrations were used as an overall marker of prenatal exposure.

We conducted PCB assays using routine methods with the same quality control procedures as previously reported (Gammon et al. 2002) using the method of Brock et al. (1996). Serum organochlorine levels of 24 PCB congeners (15, 28, 56, 66, 74, 82, 99, 101, 105, 118, 138, 146, 153, 156, 167, 170, 174, 177, 178, 180, 183, 187, 199, 203) along with *p*,*p* ′ -DDE and *p*,*p* ′ -DDT were obtained. Detection limits were 0.2 μg/L for DDE and DDT and 0.07 μg/L for PCB congeners. When the serum pool and blanks were considered together, the limit of detection for the PCB congeners was 0.01–0.1 μg/L. Congeners measured below the detection limit were assigned a value of zero. At least one PCB was detectable in 100% of our samples, although not all had detectable levels of every congener. Two PCB congeners (177 and 178) had median values below the detection limits, and one congener (82) is not persistent and did not give reliable measurements; they were excluded from analysis, and we report on the results of the 21 congeners with detectable concentrations. Serum triglycerides and cholesterol were determined by a commercial laboratory (Nichols Institute/Quest Diagnostics, Teterboro, NJ). We adjusted for normal intraindividual variations in serum PCB concentrations arising from fluctuations in serum lipid concentrations by including serum cholesterol and triglyceride concentrations as independent variables in all statistical analyses (Koopman-Esseboom et al. 1996).

Of the 162 children in the study, PCB levels were available for 154. After excluding twins, our final sample size was 150 (109 boys and 41 girls).

### Classification of exposure variables.

Maternal sera PCB concentrations were aggregated into group measures based on the degree of *ortho*-substitution of each PCB congener. We measured concentrations of one non-*ortho*-substituted PCB (PCB-15), eight mono-*ortho*-substituted PCBs (congeners 28, 56, 66, 74, 105, 118, 156, 167), seven di-*ortho*-substituted PCBs (congeners 99, 101, 138, 146, 153, 170, 180), and five tri-*ortho*-substituted PCBs (congeners 174, 183, 187, 199, 203). We did not measure any non-*ortho*-substituted PCBs that are dioxin-like. Exposure to each of four groupings of PCBs, categorized by degree of *ortho*-substitution, was analyzed in separate models. We performed secondary analyses using the individual PCB congeners as the principal exposure variable in separate regression models.

### Selection of potential covariates.

We chose as potential covariates those variables measured and available in the NCPP files that are known or suspected from previous research to influence the growth of children and that could plausibly be associated with PCB exposure. These included maternal sera concentrations of DDE, gestational age at birth, mother’s age at birth, mother’s height and prepregnancy weight, parity, maternal smoking during pregnancy, breast-feeding (yes/no), and markers of socioeconomic status, including a socioeconomic index, income at registration, and maternal years of education.

### Statistical analyses.

We used linear regression with repeated measures in our primary analyses. Before conducting these analyses, we performed separate linear regression analyses of height and weight at four ages (birth and 4, 7, and 17 years of age) to assess whether any association between exposure and outcome was limited to a specific time point. Height and weight were modeled separately, instead of with a grouped measure such as body mass index, to investigate whether PCB exposure influences height and weight differently. We performed separate analyses for each of the four grouped measures of PCB concentrations classified by degree of *ortho*-substitution. We then used linear regression with repeated measures to describe the association between maternal concentrations of PCBs, grouped by degree of *ortho*-substitution, and child’s height and weight through 17 years of age. We used generalized estimating equations (Liang and Zeger 1986; Zeger and Liang 1986) to estimate regression parameters, which modeled the overall association between PCB exposure during pregnancy and postnatal height and weight, accounting for within-subject correlation. Regressions with repeated measures were performed both including and excluding data from 17 years of age to assess the associations between prenatal PCB exposure and height and weight before and after the onset of puberty. In an additional set of analyses to discern associations between concentrations of individual PCB congeners and height and weight, we performed repeated-measures linear regressions using maternal concentrations of individual PCB congeners as exposure measures, standardized to a mean of zero and a standard deviation of one.

To satisfy the statistical requirements of the models, we applied logarithm transformations to all outcome variables to stabilize their variances. Predictor variables (including the PCB measures, serum triglyceride and cholesterol, and maternal height and weight) with skewed distributions were also log-transformed. For all regression analyses, the basic model included the PCB variable of interest as well as serum cholesterol and triglyceride levels. Covariates associated with the outcomes that changed estimate of the regression coefficient of the PCB variable on any of the height and weight outcomes by > 50% of a SE were included in the final regression model. Mother’s prepregnancy weight, preterm delivery, and mother’s height (for the children’s height regression analyses) were retained in final models. The presence of neurologic soft signs at 7 years of age—the original selection factor for the cohort under investigation—did not change the regression parameter estimates or their precision and was therefore not included in the final models. In addition, blood concentrations of DDE, although correlated with PCB levels, were not associated with the outcome measures under investigation and therefore did not influence the magnitude of the PCB–height or PCB–weight association obtained through regression analyses.

## Results

Table 1 shows baseline characteristics of our study sample and those of the entire Columbia-Presbyterian NCPP cohort. Our sample was similar to the Columbia-Presbyterian NCPP cohort with respect to a variety of characteristics thought or suspected to influence childhood growth, including gestational age at delivery, mother’s age, mother’s height and weight, and birth weight. There were proportionately more boys in our sample (73%) than in the overall Columbia-Presbyterian NCPP cohort (52%) because of higher prevalence of neurologic soft signs in boys.

Table 2 provides the distribution of maternal concentrations of PCBs in our sample, and Table 3 provides baseline characteristics of the study population stratified by quartiles of total maternal PCB concentrations. As expected, older mothers had higher concentrations of PCBs, reflecting cumulative exposure with age. Most other baseline variables were similar among the exposure categorizations.

### Regression analyses.

Table 4 presents the regression coefficients and 95% confidence intervals (CIs) from the “age-specific” analyses modeling the association between maternal levels of PCBs and weight (or height) separately for each measured time point, whereas Table 5 presents the same results from the repeated measures analyses. The regression coefficients in Table 4 correspond to the unit change in the natural log of weight (grams) or height (centimeters) at each age corresponding to a one-unit change in the natural log of maternal PCB concentration (micrograms per liter). Table 5 presents the estimated regression coefficients and 95% CIs from the repeated-measures analyses modeling the association between maternal levels of PCBs and weight (or height) measured at birth and at 4, 7, and 17 years of age. The regression coefficient (*b*) in Table 5 was related to the quantity (0.693*b*) that corresponds to the average change in the natural log of weight (grams) [or height (centimeters)] at each age used in the analysis for a doubling of maternal PCB concentrations.

#### Girls’ weight.

Concentrations of *ortho*-substituted PCBs were associated with reduced girls’ weight, particularly at 4 and 7 years of age, with no observable pattern in the magnitude of association between groups with different degrees of *ortho*-substitution (Table 4). In contrast, the non-dioxin-like non-*ortho*-substituted PCB-15 was not associated with girls’ weight at any measured time point. To put the magnitude of the regression coefficients in perspective, a doubling of maternal levels of mono-*ortho*-substituted PCBs was associated with an 11% reduction in weight measured at 4 and 7 years, after adjustment for maternal prepregnancy weight, preterm status, and serum cholesterol and triglyceride levels. The magnitude of association was similar, but slightly less, when using the exposure groups with greater degrees of *ortho*-substitution.

Repeated-measures analyses of the relationship between maternal concentrations of PCBs and girls’ weight through 17 years of age showed that concentrations of *ortho*-substituted PCBs were associated with reduced girls’ weight through 17 years of age, whereas the non-*ortho*-substituted PCB was not associated with girls’ weight (Table 5).

#### Boys’ weight.

Maternal levels of di-*ortho*-substituted PCBs were marginally associated with increased boys’ weight at 4 but not at 7 years of age (Table 4). No other categorizations were associated with boys’ weight, although the direction of the regression coefficients was opposite that found in the girls’ analyses. The magnitudes of the estimated regression coefficients were small compared with those observed among girls and not appreciably different from zero in most situations.

Repeated-measures analysis through 17 years of age showed no association between PCB exposure and boys’ weight (Table 5).

#### Girls’ height.

Maternal concentrations of the non-dioxin-like, non-*ortho*-substituted PCB (PCB-15) were associated with increased girls’ height at 4, 7, and 17 years of age, whereas exposure to *ortho*-substituted PCBs was not associated with girls’ height (Table 4). Among girls, a doubling of maternal concentrations of the coplanar PCB-15 was associated with a 2% increase in height at 4 and 7 years of age.

When the data were analyzed using linear models with repeated measures through 17 years of age, similar patterns were found (Table 5). In particular, the non-*ortho*-substituted PCB-15 was associated with increased girls’ height through 17 years of age, whereas the summed measure of mono-*ortho*-substituted PCBs was inversely, but nonsignificantly, associated with girls’ height. No associations were found when using the higher-*ortho*-substituted summary measures or the overall sum of PCBs as the exposure variable.

#### Boys’ height.

Boys’ height was not associated with maternal PCB concentrations at any of the time points measured (Table 4). The direction of the regression coefficients was opposite that seen in the girls’ analyses, although their magnitudes were not appreciably different from zero. When the data were analyzed using linear regression with repeated measures through 17 years of age, exposure to tri-*ortho*-substituted PCBs was associated with a small increase in height, with no other exposure categorizations showing an association.

### Secondary analyses.

We conducted a secondary repeated-measures analysis excluding from the model data at 17 years of age to investigate associations before the onset of puberty. This created marginally stronger-magnitude associations between exposure to *ortho*-substituted PCBs and girls’ weight (data not shown). This was expected because the strongest associations between PCB exposure and outcome occurred at 4 and 7 years of age. No other results were substantially changed.

**Figure 1:**
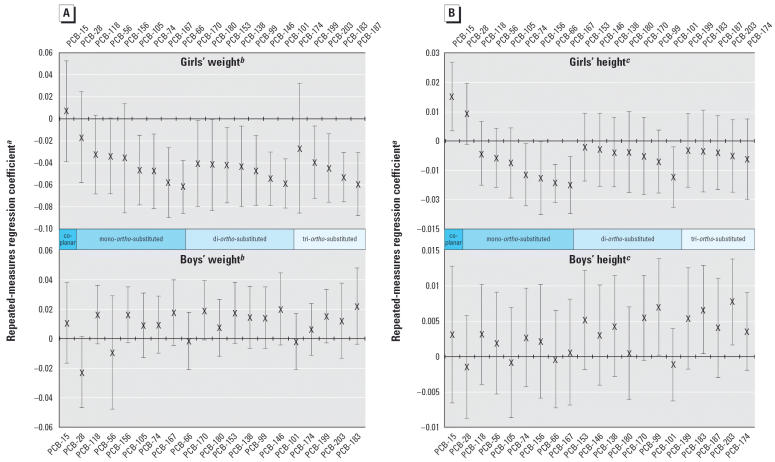
Congener-specific repeated measures regression: weight (g) (*A*) and height (cm) (*B*) through age 17 years of age vs. prenatal PCB exposure (μg/L). ***a***Beta coefficients and 95% CIs are obtained after standardizing the PCB measure to a mean of 0 and standard deviation of 1. ***b***Weight and PCB measures were log-transformed, such that the beta coefficients correspond to the change in the natural log of weight (g) associated with a standard deviation increase in the natural log of the PCB measure, adjusted for mother’s prepregnancy weight, preterm delivery, and serum cholesterol and triglyeride levels. ***c***Height and PCB measures were log-transformed, such that the beta coefficients correspond to the change in the natural log of height (cm) associated with a standard deviation increase in the natural log of the PCB measure, adjusted for mother’s prepregnancy weight and height, preterm delivery, and serum cholesterol and triglyceride levels.

To assess whether specific PCB congeners had a large influence on height or weight, we performed separate repeated-measures analyses for the 21 PCB congeners. Figure 1A presents the results of the congener-specific linear regression analyses using weight as the outcome. Fifteen of 20 *ortho*-substituted PCBs were significantly associated with reduced weight through 17 years of age in girls. In contrast, among boys, none of the PCB con-geners was significantly associated with weight through 17 years of age, and nearly all had a different direction of association than was found among girls.

Reduced girls’ height was associated with exposure to four of the eight measured mono-*ortho*-substituted PCBs and one of the seven di-*ortho*-substituted PCBs but not to higher-*ortho*-substituted PCBs (Figure 1B). Increased girls’ height was associated with exposure to the non-dioxin-like but coplanar PCB-15 and the mono-*ortho*-substituted PCB-28. In contrast, exposure to one di-*ortho*-substituted and two tri-*ortho*-substituted PCBs was marginally associated with increased height among boys. This analysis did not have sufficient power to discern whether the magnitudes of associations were significantly different among congeners having the same degree of *ortho*-substitution.

Additional analyses investigated the robustness of the results. First, as noted above, the cohort from which this sample was derived was originally selected on the basis of the presence of neurologic soft signs at 7 years of age. Adding an indicator variable for the presence or absence of soft signs to the regression models did not change the results. Second, we conducted a post hoc analysis to assess whether including the 12-month height and weight measures (which were excluded from primary analyses) influenced the results. Including this time point did not influence the magnitude or precision of the repeated-measures analyses (data not shown).

## Discussion

Prenatal exposure to *ortho*-substituted PCBs during pregnancy was associated with reduced girls’ but not boys’ weight up to 17 years of age in this study. Associations between prenatal PCB exposure and height were less consistent, with exposure to the only measured non-*ortho*-substituted PCB associated with increased height among girls, and exposure to tri-*ortho*-substituted PCB levels associated with increased height through 17 years of age among boys, although the small magnitude of this association questions the relevance of this finding. The potential for PCBs to influence growth differentially by sex needs to be further investigated in studies with larger sample sizes to statistically test for potential sex interactions.

Our findings are in general agreement with some, but not all, previous epidemiologic studies. PCB exposure was more strongly associated with reduced growth among girls in the Japanese and Taiwan cohorts (Guo et al. 1994, 1995; Rogan et al. 1988; Yamashita and Hayashi 1985) as well as the Michigan cohort (Blanck et al. 2002; Jacobson et al. 1990). Our findings differ from those reported in the North Carolina study, which found no evidence of an association between prenatal PCB exposure and height or weight through 14 years of age (Gladen et al. 2000), and from those reported by Karmaus et al. (2002), which found reductions in girls’ growth through 8 years of age associated with childhood exposure to DDE but not to PCBs. Possible reasons for the divergence between this study and those cited here may center on differences in exposure and population characteristics. Our study measured prenatal exposure to PCBs in an urban population with a low prevalence of ever breast-feeding (16%), whereas the North Carolina study population had a much higher level of ever breast-feeding (89%) (Rogan and Gladen 1991), and the measures in the Karmaus et al. (2002) study were taken at 8 years of age. It is possible that prenatal exposures to PCBs have different effects on growth than do childhood exposures or exposures via breast-feeding. For example, investigations in a Dutch cohort found an association between prenatal PCB exposure and growth rate through 3 months of age among formula-fed but not breast-fed infants, which the authors speculated may be due in part to the beneficial effects of breast-feeding acting to counter the putative effects of PCB exposure (Patandin et al. 1999). Our study is the first to examine the association between prenatal PCB exposure and height and weight through 17 years of age.

We chose not to categorize the PCBs available using toxic equivalency factors (TEFs) (Van den Berg et al. 1998), as has been done in previous analyses (e.g., Patandin et al. 1998). First, the TEFs of PCBs are based on their relative “dioxin-like” binding affinity to the aryl hydrocarbon receptor (AhR). Our hypothesis was that PCBs might influence height or weight through mechanisms other than an AhR-mediated one. Second, of the PCBs measured in our analysis, only four have an assigned TEF based on dioxin-like activity (Van den Berg et al. 1998). These PCBs (congeners 105, 118, 156, 167) have very low dioxin-like activity, with the TEFs ranging from 0.00001 (PCB-167) to 0.0005 (PCB-156). Because our *a priori* hypothesis was focused on a non-dioxin-like effect of PCBs based on structure, we felt that a classification scheme based on structure was appropriate.

Our study has potential limitations. First, the sample size was relatively small. However, we found evidence of an association between prenatal exposure to *ortho*-substituted PCBs and girls’ weight despite the small sample size. This strengthens the evidence of a true association between these variables. We had more than twice as many boys as girls in our sample and found little evidence of an association between maternal PCB levels and boys’ weight. The results of the height analyses, which showed some evidence of a positive effect of exposure to some tri-*ortho*-substituted PCBs on boys’ height, were also limited because of sample size.

Our ability to measure specific PCB congeners led to the potential problem of multiple statistical tests, which may increase the chance of a type I error. However, our analysis is concerned primarily with the grouped measures of exposure based on the degree of *ortho*-substitution. Thus, rather than attempting to look at the statistical significance of every analysis performed, more can be gained by looking for patterns. The facts that the direction of association was consistently different between boys and girls and that the association with weight was consistently stronger than with height provide support that the associations observed here were not simply due to multiple comparisons.

Height and weight measurements were also subject to measurement error. Evidence in the NCPP data files from which the height and weight measures were obtained showed that the weight measurement at 17 years of age may have slight errors due to an improperly functioning scale. Because of this, the secondary analyses excluding 17 years of age data from the repeated-measures analysis served the dual purposes of eliminating the error-prone 17-year measures as well as confining our height and weight analysis to prepubertal age points. Differences between the analyses including and excluding the 17-year measures were discussed, and the interpretation of the results does not change in any material way after exclusion of the 17-year measures. There is no reason to suspect that these measurement errors were differential based on exposure or outcome status.

Although the NCPP data files contain extensive information on many variables thought to be associated with childhood growth, it is of course not exhaustive. We had no information among the individuals in this study on age at puberty, which could be useful as an outcome for further study. We also did not have information on specific measures of growth (e.g., leg growth, whose growth spurt occurs predominantly before puberty), which precluded us from assessing specific aspects of growth that may have been affected by prenatal exposure to PCBs. Further, we also did not have sufficient sera available to measure levels of other environmental contaminants, such as dioxin-like PCBs, dioxin, lead, or mercury. Because of this, we are unable to rule out the possibility that the associations seen in this study are due partly to confounding by these other environmental contaminants. However, because exposure to *ortho*-substituted PCBs was associated with weight but not height, and in girls but not boys, such unmeasured confounding would have to operate only among girls and only for weight to explain these results.

Our underlying hypothesis is a biologic one: that prenatal exposure to specific types of PCBs adversely affects endocrine systems in children, which can then lead to reductions in height and weight. If one of the causal mechanisms through which PCB exposure acts is via interference with normal sex hormone processes, then a finding specific to girls would not be unexpected. Our analysis could not discern whether different degrees of *ortho*-substitution were more associated with reduced weight measures. The results of the analyses using height as the outcome are more equivocal and have a much smaller magnitude of association than that seen for the weight analyses. The only non-*ortho*-substituted PCB measured in this analysis was associated with increased height measures among girls, whereas certain mono-*ortho*-substituted PCBs were associated with decreased height, and the higher-*ortho*-substituted PCBs showed no association with girls’ height. Among boys, certain tri-*ortho*-substituted PCBs were associated with increased height, whereas most were not associated with height. The lack of a clear trend of association, as was seen in the weight analyses, places interpretations from the height analyses as speculative.

These findings, limited by measurement error and a small sample size, suggest an interesting direction for future research. Animal and laboratory evidence strongly suggests that the structure of PCB congeners determines their biologic effect. This study provides evidence that *ortho*-substituted PCBs are associated with reduced weight in girls but not in boys. Further studies are needed to clarify this relationship.

## Figures and Tables

**Table 1:** Characteristics of the study population compared with the entire Columbia-Presbyterian NCPP study population.

Characteristics	Columbia-Presbyterian NCPP cohort (*n* = 2,177)	Our study population (*n* = 150)
Sex (% male)	52	73
Birth weight (g)	3,178 ± 957	3,096 ± 555
Birth length (cm)	50 ± 3.6	50 ± 2.8
Gestational age at delivery (weeks)	39.0 ± 5.3	38.5 ± 3.4
5-Min Apgar score	8.7 ± 1.4	8.5 ± 1.5
Parity	2.1 ± 1.6	2.0 ± 1.5
Maternal age (years)	25.7 ± 5.9	25.9 ± 6.2
Maternal prepregnancy weight (kg)	58.5 ± 10.9	60.8 ± 11.3
Maternal height (cm)	163 ± 15	165 ± 8
Maternal smoking during pregnancy (%)	38	24
Maternal years of education	10.5 ± 2.6	11.4 ± 1.9
Mothers married at registration (%)	88	75

Values are mean ± SD except where otherwise noted.

**Table 2:** Distribution of maternal PCB concentrations (μg/L).

		Percentile distributions			
Exposure category	No.	25	50	75	Mean ± SD
∑PCB_all_^a^	150	6.8	8.4	10.4	9.2 ± 3.5
PCB-15 (coplanar)	135	0.5	1.0	1.7	1.2 ± 0.8
∑PCB_mono_^b^	150	2.3	2.8	3.5	3.1 ± 1.3
∑PCB_di_^c^	150	3.0	3.6	5.0	4.2 ± 2.0
∑PCB_tri_^d^	150	0.5	0.7	1.0	0.8 ± 0.5

Abbreviations: ∑PCB_all_, sum of all PCBs; ∑PCB_di_, sum of di-*ortho*-substituted PCBs; ∑PCB_mono_, sum of mono-*ortho*-substituted PCBs; ∑PCB_tri_, sum of tri-*ortho*-substituted PCBs.

a PCB congeners summed into ∑PCB_all_: 15, 28, 56, 66, 74, 99, 101, 105, 118, 138, 146, 153, 156, 167, 170, 174, 180, 183, 187, 199, 203.

b PCB congeners summed into PCB_mono_: 28, 56, 66, 74, 105, 118, 156, 167.

c PCB congeners summed into PCB_di_: 99, 101, 138, 146, 153, 170, 180.

d PCB congeners summed into PCB_tr_: 174, 183, 187, 199, 203.

**Table 3:** Baseline and outcome characteristics by quartile group of total PCB exposure.

	Quartile groups of total PCB exposure			
Quartile group [μg/L (mean PCB level)]	1 (5.8)	2 (7.7)	3 (9.4)	4 (13.8)
Total sample (*n* = 150)				
Birth weight (g)	3,202 ± 578	3,010 ± 551	3,033 ± 477	3,129 ± 615
Birth length (cm)	50.0 ± 2.5	49.3 ± 3.4	49.1 ± 2.5	49.6 ± 2.9
Gestational age at delivery (weeks)	39.2 ± 2.6	38.6 ± 3.6	37.9 ± 4.0	38.5 ± 3.2
Parity	1.7 ± 1.2	1.9 ± 1.3	2.6 ± 2.0	1.8 ± 1.1
Maternal age (years)	23.9 ± 6.1	25.6 ± 6.1	26.5 ± 6.2	27.6 ± 6.1
Maternal prepregnancy weight (lbs)	138 ± 30	132 ± 26	139 ± 26	128 ± 17
Maternal height (in)	64 ± 3	65 ± 3	65 ± 2	64 ± 2
Maternal smoking during pregnancy (%)	41	43	41	42
Maternal years of education	11.4 ± 1.4	11.6 ± 1.9	10.9 ± 2.5	11.3 ± 2.1
Family income at registration ($)	4,019 ± 1,673	4,379 ± 2,261	5,067 ± 2,373	5,719 ± 2,624
Socioeconomic index at registration	59.8 ± 19.2	51.3 ± 21.6	54.3 ± 18.3	55.5 ± 15.4
Girls (*n* = 41)				
Birth weight (g)	3,050 ± 786	3,064 ± 470	3,070 ± 403	3,082 ± 499
Birth length (cm)	49.3 ± 3.2	49.0 ± 2.0	49.0 ± 2.7	49.3 ± 2.2
Gestational age at delivery (weeks)	38.5 ± 3.2	38.7 ± 2.8	39.3 ± 1.5	39.0 ± 0.9
Parity	2.0 ± 1.0	2.0 ± 1.8	3.4 ± 3.0	2.1 ± 0.9
Maternal age (years)	27.3 ± 7.0	25.8 ± 7.5	29.9 ± 6.1	29.3 ± 5.6
Maternal prepregnancy weight (lbs)	139 ± 23	135 ± 22	150 ± 30	132 ± 10
Maternal height (in)	64 ± 2	66 ± 2	65 ± 2	64 ± 2
Maternal smoking during pregnancy (%)	30	36	71	30
Maternal years of education	12.3 ± 1.4	11.4 ± 2.4	10.9 ± 1.7	11.0 ± 2.5
Family income at registration ($)	4,333 ± 1,472	4,722 ± 2,539	5,278 ± 1,394	5,398 ± 2,619
Socioeconomic index at registration	48.4 ± 16.7	48.9 ± 25.0	55.6 ± 20.1	54.1 ± 19.6
Boys (*n* = 109)				
Birth weight (g)	3,256 ± 440	3,035 ± 623	2,898 ± 589	3,195 ± 579
Birth length (cm)	50.2 ± 2.0	49.9 ± 4.0	48.6 ± 3.2	50.0 ± 2.6
Gestational age at delivery (weeks)	39.3 ± 2.0	39.0 ± 4.4	36.7 ± 4.7	38.6 ± 3.0
Parity	1.5 ± 1.2	1.6 ± 0.9	2.2 ± 1.3	1.6 ± 1.2
Maternal age (years)	22.2 ± 5.2	25.1 ± 5.3	26.3 ± 6.9	27.2 ± 6.1
Maternal prepregnancy weight (kg)	60.3 ± 15.0	59.9 ± 15.2	63.0 ± 11.3	58.1± 8.2
Maternal height (cm)	163 ± 8	163 ± 8	165 ± 5	165 ± 5
Maternal smoking during pregnancy (%)	42	48	36	48
Maternal years of education	11.2 ± 1.3	11.7 ± 1.6	10.9 ± 2.8	11.5 ± 1.9
Family income at registration ($)	3,929 ± 1,748	4,250 ± 2,192	4,976 ± 2,713	5,848 ± 2,673
Socioeconomic index at registration	50.2 ± 20.2	52.4 ± 20.2	53.7 ± 18.0	56.0 ± 13.7

Values are mean ± SD except where otherwise noted.

**Table 4:** Results of simple linear regression: weight and height versus prenatal PCB exposure, by sex.^a^

	Weight [parameter estimate (95% CI)]^b^				Height [parameter estimate (95% CI)]^b^			
Exposure^c^	Birth	4 years	7 years	17 years	Birth	4 years	7 years	17 years
Girls								
PCB-15 (coplanar)	0.07 (–0.03 to 0.17)	0.01 (–0.06 to 0.08)	–0.03 (–0.11 to 0.06)	–0.01 (–0.12 to 0.09)	0.02 (–0.01 to 0.05)	0.02 (0.01 to 0.04)	0.02 (0.00 to 0.04)	0.02 (0.00 to 0.04)
∑PCB_mono_	–0.16 (–0.31 to –0.01)	–0.17 (–0.26 to –0.07)	–0.16 (–0.31 to –0.02)	–0.11 (–0.27 to 0.06)	–0.04 (–0.08 to 0.01)	–0.01 (–0.04 to 0.03)	–0.01 (–0.05 to 0.02)	–0.02 (–0.06 to 0.01)
∑PCB_di_	–0.09 (–0.22 to 0.05)	–0.15 (–0.23 to –0.06)	–0.15 (–0.27 to –0.03)	–0.06 (–0.20 to 0.08)	–0.01 (–0.05 to 0.03)	0.00 (–0.04 to 0.03)	–0.01 (–0.04 to 0.02)	–0.02 (–0.05 to 0.01)
∑PCB_tri_	–0.03 (–0.15 to 0.08)	–0.12 (–0.19 to –0.05)	–0.15 (–0.25 to –0.05)	–0.08 (–0.18 to 0.03)	0.00 (–0.04 to 0.03)	0.00 (–0.03 to 0.02)	–0.02 (–0.04 to 0.01)	–0.02 (–0.04 to 0.01)
∑PCB_all_	–0.09 (–0.26 to 0.08)	–0.18 (–0.28 to –0.07)	–0.20 (–0.35 to –0.04)	–0.10 (–0.27 to 0.07)	–0.02 (–0.06 to 0.03)	0.01 (–0.03 to 0.05)	0.00 (–0.04 to 0.04)	–0.02 (–0.06 to 0.02)
Boys								
PCB-15 (coplanar)	0.00 (–0.03 to 0.02)^b^	0.01 (–0.01 to 0.03)	0.01 (–0.02 to 0.03)	0.02 (–0.01 to 0.05)	0.00 (–0.01 to 0.01)	0.00 (0.00 to 0.00)	0.00 (0.00 to 0.01)	0.00 (0.00 to 0.01)
∑PCB_mono_	0.02 (–0.07 to 0.11)	0.02 (–0.05 to 0.09)	0.01 (–0.08 to 0.09)	–0.03 (–0.16 to 0.10)	0.00 (–0.02 to 0.03)	–0.01 (–0.03 to 0.02)	0.01 (–0.01 to 0.02)	0.00 (–0.02 to 0.03)
∑PCB_di_	0.03 (–0.05 to 0.12)	0.06 (0.00 to 0.13)	0.05 (–0.03 to 0.13)	0.00 (–0.12 to 0.12)	0.00 (–0.02 to 0.03)	0.01 (–0.02 to 0.03)	0.01 (–0.01 to 0.03)	0.00 (–0.02 to 0.03)
∑PCB_tri_	0.01 (–0.05 to 0.07)	0.03 (–0.02 to 0.08)	0.02 (–0.04 to 0.07)	–0.02 (–0.10 to 0.07)	0.00 (–0.02 to 0.02)	0.01 (0.00 to 0.03)	0.01 (0.00 to 0.02)	0.01 (–0.01 to 0.02)
∑PCB_all_	0.00 (–0.10 to 0.10)	0.04 (–0.03 to 0.12)	0.03 (–0.06 to 0.12)	–0.02 (–0.16 to 0.13)	0.00 (–0.06 to 0.03)	0.01 (–0.03 to 0.05)	0.00 (–0.04 to 0.04)	–0.02 (–0.06 to 0.02)

Abbreviations: ∑PCB_all_, sum of all PCBs; ∑PCB_di_, sum of di-*ortho*-substituted PCBs; ∑PCB_mono_, sum of mono-*ortho*-substituted PCBs; ∑PCB_tri_, sum of tri-*ortho*-substituted PCBs.

a Parameter estimates for the log-transformed PCB measure, adjusted for maternal prepregnancy weight, preterm status, and serum triglyceride and cholesterol. Models with height as the outcome were also adjusted for maternal height.

b Height and weight measures were log-transformed; regression parameters present the unit change in ln[weight (g)] or ln[height (cm)] for a one-unit change in ln[PCB (μg/L)].

c All PCB exposure measures were summed by structural categorization (non-, mono-, di-, or tri-*ortho*-substituted, or sum of all PCB measures) and then log-transformed.

**Table 5:** Estimated coefficients (95% CIs) for regression models with repeated measures: birth and 4, 7, and 17 years of age.

	Outcome^a^			
Exposure^b^	Girls’ weight	Girls’ height	Boys’ weight	Boys’ height
PCB-15 (coplanar)	1.1 (–5.9 to 8.1)	1.9 (–0.0 to 3.8)	0.7 (–1.1 to 2.4)	0.2 (–0.4 to 0.7)
∑PCB_mono_	–13.7 (–20.8 to –6.6)	–1.8 (–4.2 to 0.6)	0.6 (–4.9 to 6.0)	0.0 (–1.7 to 1.8)
∑PCB_di_	–10.6 (–18.2 to –3.0)	–1.1 (–3.5 to 1.4)	3.7 (–1.6 to 9.0)	0.6 (–0.9 to 2.1)
∑PCB_tri_	–9.2 (–15.0 to –3.3)	–1.0 (–3.1 to 1.2)	1.8 (–2.0 to 5.6)	1.2 (0.1 to 2.2)
∑PCB_all_	–13.2 (–20.7 to –5.7)	–0.6 (–3.2 to 2.0)	1.7 (–4.7 to 8.1)	0.5 (–1.4 to 2.3)

Abbreviations: ∑PCB_all_, sum of all PCBs; ∑PCB_di_, sum of di-*ortho*-substituted PCBs; ∑PCB_mono_, sum of mono-*ortho*-substituted PCBs; ∑PCB_tri_, sum of tri-*ortho*-substituted PCBs. Data are 100 × the parameter estimates for the log-transformed PCB measure, adjusted for maternal prepregnancy weight, preterm status, and serum triglyceride and cholesterol. Models with height as the outcome were also adjusted for maternal height.

a Height and weight measures were log-transformed.

b All PCB exposure measures were summed by structural categorization and then log-transformed.
